# Three Cases of First Labor in Women with Congenital Dislocation of One Hip*Read before the Wayne County Medical Society, September 30, 1918.

**Published:** 1918-12

**Authors:** Mary G. Haskins

**Affiliations:** Detroit, Mich.


### Three Cases of First Labor in Women With Congenital Dislocation of One Hip.*

By MARY G. HASKINS, M. D., Detroit.

Dislocation of one hip as a complication of pregnancy and labor is treated very casually in the text-books, so that one concludes that either the condition is not frequently met or that it is no serious menace to a patient during labor.

However, the presence on ones waiting list of a patient with so obvious a deformity is a stimulus to study of the case and of the literature.

These three cases add little to what has already been noted but they are perhaps of interest because the changes in the pelves are so uniform and are due in each case to exactly the same displacement of the same hip and that all are free from complications.

It is interesting to find that most of the cases reported are grouped with cases of oblique pelvis from other causes, notably infantile paralysis and hip-disease. For all practical purposes this is probably satisfactory, but the findings in these three cases are the effect of uncomplicated, unilateral, congenital dislocation of the hip.

Case 1. Mrs. B. was sent to me by a doctor up in the state who does a large country obstetrical practice. He did not make a diagnosis but decided from her appearance that hospital care would be necessary and sent her down with her mind prepared for a Caesarean section.

She was not preposessing to look at; it was not surprising that he did not want to tackle her in a lonely farm house without help and in the poorest of surroundings.

She was short and squat in figure with unmistakable signs of early rickets, with rolls of fat around the waist and hips, with a pendulous abdomen that was thrust violently forward at every step on the shortened leg, she was 42 years of age, pregnant for the first time and was within one week of the estimated date of delivery.

The family history was negative and the personal history meager. She was a perfectly strong, well baby. The parents were poor, the family was large and the home was far in the country, so, as the little girl did not suffer at all, the lameness which developed as soon as she began to walk was treated as a dispensation of Providence and received no medical attention whatever.

Owing to the amount of fat overlying the pelvic bones it was impossible to determine accurately the position of the head of the femur or to take very satisfactory pelvic measurements, but it was evident that the pelvis was if anything a

*Read before the Wayne County Medical Society, September 30, 1918.

little oversize and there could be made out a difference of nearly 3 c.m. in the oblique diameters.

There was no fixation of the hip-joint, no limitation of motion and no lordosis.

There was a marked lumbo-sacral curve with a corresponding sidewise tilt of the pelvis, but as there was a difference of nearly 3 inches in the length of the legs, measured from the anterior superior spines to the internal malleoli, it seemed that the scoliosis was more effect than cause.

The promontory could not be reached by internal examination, but it seemed that this might be due to the difficulty in examination on account of the excessive fat, for the head was floating above the brim in this large pelvis with the patient at term so there must have been some shortening of the antero-posterior diameter.

The diagnosis of congenital dislocation of one hip was made from

1—the history of lameness from birth without disease,
2—the shortened leg without ankylosis,
3—the peculiar thrusting gait.

Two skiagraphs were made, one to confirm the position of the head of the femur and the other to determine if possible what effect the deformity was likely to have on the labor. This last was taken from the front, downward and backward, perpendicular to the plane of the inlet, in the effort to find out the amount of oblique contraction and the amount of encroachment of the promontory. They were taken at Providence Hospital when the apparatus was new and the operator unaccustomed to it and they were unfortunately very poor. The head of the femur could be made out displaced upward and backward, confirming the diagnosis, but the other plate was worthless, so the choice of method of delivering the patient had to be made without its help.

Some changes however could be deduced. The pressure of one femoral head upon the upper and back part of the innominate bone must of necessity tilt the sacrum forward, thereby narrowing the inlet and widening the outlet anteroposteriorly. The unequal pressure of the two femora must result in an oblique contraction of the pelvis and a spreading of the tuberosities, while the scoliosis, although secondary to the shortened leg, must during its development bring about a rotation of the sacrum on its long axis.

The forces which had operated on the bony girdle seemed than to have brought about a mixture of conditions which, if one might coin a name, might best be described as a rachitic, scolio-coxalgic pelvis without ankylosis.

Since the measurements were somewhat above the average and since neither the flattening nor the obliquity was likely to make trouble unless excessive, it was decided, in spite of her age, to bring on the labor and let the patient try to deliver herself.

A bougie was introduced and the cervix packed with gauze. Pains came on which were annoying but not productive and in 48 hours the procedure was repeated, this time with good results.

Dilatation occupied 36 hours but as the head engaged and became well fixed during that time, and as the patient was in the hospital and the family under control, she was allowed to go on without interference.

After the rupture of membranes the head was 4 hours in descending and rotating but at no time was there a let-up in the contractions until the head was on the perineum, when they became ineffectual.

Low forceps were undoubtedly indicated, but this time I was so much interested in seeing the patient deliver herself that, although the use of pituitrin was in its early days, I gave her one ampoule, not without misgivings.

Pains recommenced and although the delivery was slow it was accomplished without forceps and with the baby in excellent condition.

The whole labor had lasted a little over 40 hours.

Case 2. Mrs. J., aged 37, and pregnant for the first time. She too had been perfectly well as a child and her lameness was noticed only as she began to walk.

This family was in a very different social class from the first one and medical advice was sought at once. The family physician made the diagnosis but advised no treatment other than keeping the patient in the best of physical condition. She never suffered from the lameness in the least except as a handicap in dancing and skating with other children.

Her measurements except for the oblique diameters were normal. Plates made by Dr. Vine Larue Smith showed the same upward and backward displacement of the head of the femur and the same spread of the tuberosities. In this case we also got a very good picture of the superior strait, showing a marked forward projection of the promontory and a moderate obliquity.

This patient was seen long enough beforehand so that she could have some preparation for her labor. She had a warm sitz bath every night and a teaspoonful of sodium phosphate every morning before breakfast for six weeks before delivery.

Either because of the treatment or in spite of it she went into labor on the exact date estimated and was delivered spontaneously after labor of 17 hours.

She too was given a dose of pituitrin when pains became ineffectual during the second stage.

Case 3. Stella H., aged 21, pregnant for the first time and lame from infancy, differs from the other cases in that she had much treatment in her childhood. Her family lived in Ann Arbor and when her lameness was discovered as she began to walk she was taken to the clinic at the University Hospital and was under treatment for over 2 years.

Several operations, one of which at least was an open operation, were undertaken for the reduction of a congenital dislocation. As a result she has a scar on the thigh, a slight adduction of the femur and a moderate stiffening of the hip-joint.

With these exceptions the findings are identical with those in case 2, as is shown in good plates made by Dr. Hickey.

Her pelvis was full size and as the baby seemed small she was allowed to go nearly 2 weeks over the estimated date for her delivery, at the end of which time labor started spontaneously and she delivered herself in 11 hours. She was given the same preliminary treatment as case 2.

Although few in number these cases cover rather a wide range;

1—They vary in age from 21 years to twice that number.

2—They vary with regard to early care from total neglect to strenuous care by experts.

3—The time of beginning labor varied from the day estimated to 2 weeks past that time.

4—The length of the labors varied from 11 hours to 40.

5—Two cases were under observation early, the other was not seen till term.

6—The weight of the baby in the first case was 7½ lbs., in the second 7 lbs. and in the third 6 lbs.

Consideration of these three cases would lead to the conclusion that in spite of the appearance of serious lameness the changes in the bony pelvis resulting from congenital dislocation of one hip are negligible so far as interference with parturition is concerned. And this corresponds with the statements in the text-books.

However, from the nature of these changes it would seem that in no particular case would one be justified in predicting so fortunate an outcome. In a patient with even a slight general contraction of the pelvis, or in one with even a slight oversize of the fetal head, labor is liable to arrest with the head above the brim, necessitating for its relief one of the severer obstetric operations, axis-traction forceps, version or Caesarean section.

In other words, in spite of the apparent ease of delivery in these cases it is not safe to generalize and every case should be the subject of careful study.

270 Woodward Ave.

